# The correlation of obesity status with serum 25-hydroxyvitamin D in US Asian adults: NHANES 2011–2018

**DOI:** 10.1371/journal.pone.0301327

**Published:** 2024-04-16

**Authors:** Linjie Qiu, Yan Ren, Jixin Li, Meijie Li, Wenjie Li, Lingli Qin, Jin Zhang, Feng Gao

**Affiliations:** 1 Prevention and Treatment Center, Xiyuan Hospital, China Academy of Chinese Medical Sciences, Beijing, China; 2 Graduate school, Shanxi University of Chinese Medicine, Shanxi, China; Kerman University of Medical Sciences Physiology Research Center, ISLAMIC REPUBLIC OF IRAN

## Abstract

**Background:**

There is a correlation between obesity and 25-hydroxyvitamin D (25OHD) that tends to be negative. However, this relationship varies among different races. In this study, Asian adults with and without obesity were compared in terms of their levels of 25OHD.

**Methods:**

We carried out a cross-sectional analysis on 2664 non-Hispanic Asian adults who participated in the National Health and Nutrition Examination Survey (NHANES) conducted between 2011 and 2018. To examine the connection between obese status, body mass index (BMI), waist circumference (WC) and weight, and 25OHD, we ran multivariate linear regression models and multivariate logistic regression models.

**Results:**

After adjusting for all confounding factors, obesity status shows a significant positive correlation with vitamin D deficiency (model 3: OR = 2.318, 95% CI:1.317, 4.082). This positive correlation remains significant in males (males: OR = 2.713, 95% CI: -13.398, 5.217). In all three models, a negative association was observed between obesity status and 25OHD (model 1: β = -4.535, 95% CI: -6.987, -2.083; model 2 β = -4.249, 95% CI: -6.549, -2.039; model 3 β = -1.734, 95% CI: -7.285, 3.816). After controlling for covariates, there was a significant negative correlation between WC and 25OHD when stratified by gender and obesity status in both males with and without obesity (males with obesity: β = -1.461, 95% CI: -2.485, -0.436; males without obesity: β = -0.855. 95% CI: -1.499, -0.210). In males with obesity, there was a very strong positive connection between body weight and 25OHD (β = 0.912, 95% CI: 0.227, 1.597). In addition, neither gender’s obese individuals showed a significant link between BMI and 25OHD.

**Conclusion:**

This study demonstrated a positive correlation between obesity and vitamin D deficiency and a negative correlation between obesity and 25OHD in Asian American adults. Additionally, among male obese individuals, there was a significant negative correlation between WC and 25OHD, an observation that needs to be validated in further prospective studies.

## Introduction

Obesity and vitamin D deficiency are two major public health concerns worldwide [1,2]. Obesity is a chronic metabolic disease that is complex and mostly linked to excessive accumulation of fat [3]. A Vitamin D is a fat-soluble vitamin that is primarily stored in adipose tissue [4], making the correlation between obesity and vitamin D deficiency a popular research topic.

Obese patients often suffer from nutrient deficiencies, including vitamin D deficiency [5]. Previous research has linked obesity to an increased risk of vitamin D insufficiency [6,7], Mahdieh et al. conducted a meta-analysis on the relationship between vitamin D and body fat mass, and found that the level of 25-hydroxyvitamin D (25OHD) was negatively correlated with body fat mass [8]. Zahra et al. conducted a systematic review of cross-sectional studies on abdominal obesity and serum vitamin D levels, and found that increased waist circumference (WC) was associated with an increased risk of vitamin D deficiency and insufficiency in adults [9]. Poonam et al. found through meta-analysis that a decrease in weight and percentage of body fat in obese subjects increased serum vitamin D levels [10], however, a prospective study conducted in Switzerland found no association between 25OHD levels and weight gain [11], and another prospective study also indicated that there was no association between vitamin D status and weight or WC gain [12]. The results of studies on the relationship between various obesity-related indicators and vitamin D levels are inconsistent. Furthermore, differences in skin color can affect vitamin D synthesis and status, potentially impacting the relationship between obesity and vitamin D across different races [13,14]. Epidemiological surveys have shown that Asian populations are more likely to experience vitamin D insufficiency compared to North American and European populations [15]. The most recent National Health and Nutrition Examination Survey (NHANES) conducted between 2011 and 2018 revealed a concerning increase in all markers of obesity among non-Hispanic Asians in the United States [2]. However, research on the relationship between obesity-related indicators and vitamin D in Asian adults is limited.

Therefore, with the aid of a nationally representative sample, this study examined the connection between vitamin D deficiency and obesity in Asian adults. We also evaluated the relationship between weight, WC, and body mass index (BMI) and vitamin D.

## Methods

### Data sources

**Fig 1:**
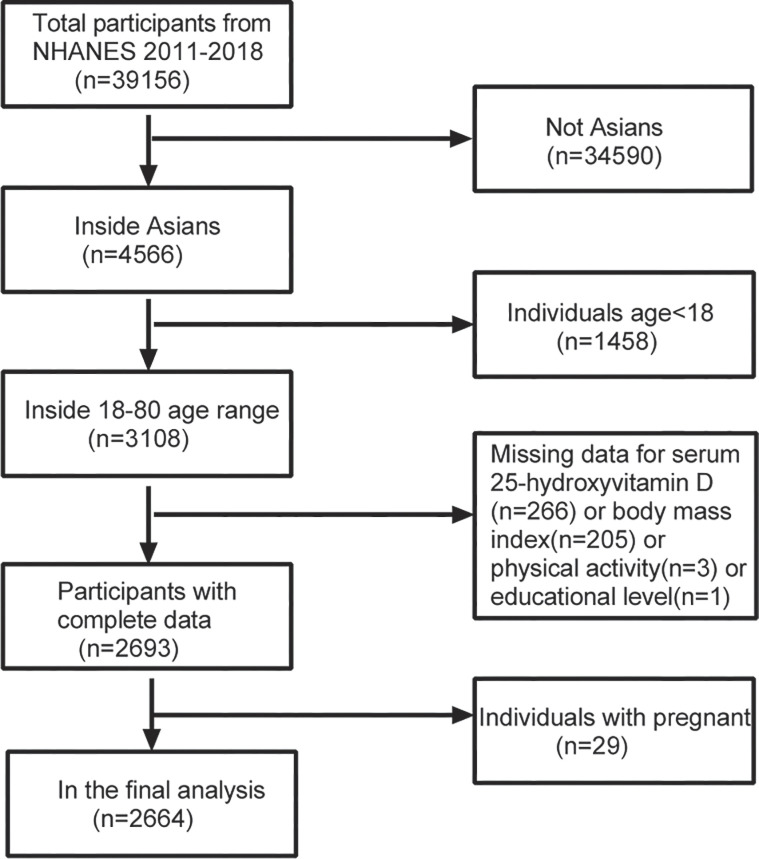
From chart of sample selection from the NHANES 2011–2018.

The National Center for Health Statistics (NCHS) conducts a nationwide survey of health and nutrition data using the NHANES, which employs a sophisticated sampling design. The survey has been conducted every two years since 1999 and collects demographic, nutritional, exam, laboratory, and questionnaire data [16,17]. Starting in 2011, NHANES began oversampling non-Hispanic Asians. For this study, we pooled survey data for a total of four cycles spanning 2011–2018. The total number of participants from 2011–2018 was 39156. We excluded 34,590 non-Asian participants as well as 1,458 individuals under the age of 18. Additionally, we excluded participants with missing 25OHD data (n = 206), BMI data (n = 205), physical activity data (n = 3), education level data (n = 1), or pregnancy status (n = 29). Ultimately, we included complete data from 2664 Asian adults for analysis (Fig 1). Detailed methodology and NHANES survey information are available on the official website. The National Center for Health Statistics’ ethical review committee approved the survey, and all participants provided their informed consent by signing a form.

### Evaluation of exposures

Exposure variables mainly included obesity status, WC, BMI and weight. Obesity was defined using the World Health Organization (WHO) ’s criteria for Asians, which states a BMI greater than or equal to 27.5 [18]. Data for WC, BMI and weight were obtained from body measurements in NHANES. Trained health technicians gathered these measurements in the Mobile Examination Center. Weight in kilograms divided by height in meters squared was used to compute BMI, which was then rounded to the nearest decimal point. The right ilium of the pelvis was located by palpating the hip region, and the waist was measured by the NHANES health technicians. The measurement was made at the participant’s typical expiration time, with the accuracy up to 0.1cm. More details could be found in the NHANES Manual for Anthropometric procedures(https://wwwn.cdc.gov/nchs/data/nhanes/2015-2016/manuals/2016_Anthropometry_Procedures_Manual.pdf).

### Outcome

This study measured the serum 25OHD level as its outcome, which is considered the most accurate indicator of vitamin D levels due to its half-life of approximately three weeks [19]. 25-hydroxyvitamin D2 (25OHD2) and 25-hydroxyvitamin D3 (25OHD3) are combined to form total 25OHD. In the test, 25OHD3 and 25OHD2 were quantitatively detected in human serum using ultra-high performance liquid chromatography-tandem mass spectrometry (UHPLC-MS/MS). The NHANES Laboratory Procedures Manual goes into great detail about how to collect and prepare samples (https://wwwn.cdc.gov/nchs/data/nhanes/2015-2016/manuals/2016_MEC_Laboratory_Procedures_Manual.pdf.) Additionally, based on recommendations from the medical research institute, we define levels of 25OHD less than 30nm/L as indicative of vitamin D deficiency, and levels of 25OHD greater than or equal to 30nm/L as indicative of non-deficiency in vitamin D [20].

### Covariates for analysis

Information on gender, age, education level, ratio of family income, and physical activity (classified as inactive, less active, or namely active as recommended by Physical Activity Guidelines) [21] were obtained from self-reported questionnaires. Detailed procedures for serum uric acid, serum sodium, glycohemoglobin, fasting glucose, total cholesterol (TC), triglycerides (TG), HDL-cholesterol (HDL-C) and LDL-cholesterol (LDL-C) were available in the NHANES website for laboratory data.

### Statistical methods

We analyzed all data statistically using the package R version 3.4.3 (http://www.R-project.org) and EmpowerStats software (http://www.empowerstats.com), and took into account sampling weights according to the NCHS-edited analytical guideline. We used means and standard deviations to report continuous data, and for categorical data, we used percentages. Statistical significance was set at 0.05. In order to determine the relationship between the presence of obesity, BMI, WC, weight, and 25OHD levels, we ran multivariate linear regression models. Furthermore, we conducted an analysis that accounted for covariates. Specifically, Model 1 did not include any adjusted covariates; Model 2 included adjustments for age and sex; and Model 3 included adjustments for all the covariates listed in Table 1. Additionally, we performed subgroup analyses based on gender and obesity status.

**Table 1:** Weighted characteristics of study sample with and without obesity.

Characteristic	Obesity(n = 671)	Non-obesity(n = 1993)	P value
Age(years)	44.60 ± 15.41	44.58 ± 16.58	0.980
Gender (%)			0.040
Male	50.62	46.08	
Female	49.38	53.92	
Educational level (%)			0.059
Less than 9th grade	6.43	6.95	
9-11th grade	6.48	7.04	
High school graduate/GED or equivalent	15.25	14.35	
Some college or AA degree	23.63	18.73	
College graduate or above	48.20	52.92	
Physical activity (%)			0.021
Inactive	71.59	76.30	
Less active	6.51	6.51	
Namely active	21.90	17.19	
Ratio of family income to poverty (%)	3.14 ± 1.64	3.16 ± 1.67	0.761
Weight(kg)	83.41 ± 14.88	61.53 ± 10.55	<0.001
Body mass index(kg/m^2^)	31.14 ± 3.81	23.02 ± 2.58	<0.001
Waist Circumference(cm)	102.13 ± 9.49	83.87 ± 8.41	<0.001
Serum uric acid (μmol/L)	357.51 ± 87.30	312.91 ± 76.91	<0.001
Serum sodium (mmol/L)	139.23 ± 2.31	139.32 ± 2.42	0.427
Glycohemoglobin (%)	5.99 ± 1.11	5.63 ± 0.83	<0.001
Fasting Glucose(mmol/L)	6.31 ± 1.58	5.77 ± 1.31	<0.001
HDL-Cholesterol (mmol/L)	1.21 ± 0.32	1.46 ± 0.41	<0.001
LDL-Cholesterol (mmol/L)	2.99 ± 0.89	2.83 ± 0.92	0.011
Triglyceride (mmol/L)	1.63 ± 1.13	1.24 ± 1.01	<0.001
Total Cholesterol (mmol/L)	5.00 ± 0.96	4.90 ± 1.04	0.032
25-hydroxyvitamin D (nmol/L)	59.36 ± 27.95	63.89 ± 28.48	<0.001

**Notes:** Mean ± SD for continuous variables: P-value was calculated by weighted linear regression model. % for categorical variables: P-value was calculated by weighted chi-square test.

## Ethics approval and consent to participate

The National Center for Health Statistics Ethics Review Committee granted ethics approval. All methods were carried out in accordance with relevant guidelines and regulations (declaration of Helsinki). All individuals provided written informed consent before participating in the study.

## Results

### Characteristics of the participants

There were differences in health data obtained between Asian obese adults and non-obese adults in terms of demographics and laboratory tests (Table 1). Male subjects with obesity were more prevalent than female subjects among Asian adults compared to their non-obese counterparts. Besides, weight, BMI, WC, serum uric acid, glycohemoglobin, fasting glucose, TC, TG, HDL-C and LDL-C were statistically different between the two groups. Notably, serum hydroxyvitamin D levels were lower in obese Asian adults than in non-obese adults of Asian descent (59.36 ± 27.95 vs 63.89 ± 28.48, p<0.001).

### Relationship between obesity status and vitamin D deficiency

As shown in Table 2, after controlling for potential confounding factors, obesity status shows a significant positive correlation with vitamin D deficiency (model 3: OR = 2.318, 95% CI:1.317, 4.082). Subgroup analysis by gender and adjustment for all confounding factors reveal that in males, this positive correlation remains significant (males: OR = 2.713, 95% CI: -13.398, 5.217), whereas in females, it is not significant (females: OR = 1.833, 95% CI: -4.607, 8.273).

**Table 2:** Association between obesity status and vitamin D deficiency.

	Model 1 OR (95%CI, P)	Model 2 OR (95%CI, P)	Model 3 OR (95%CI, P)
Non-obesity	Reference	Reference	Reference
obesity	1.344(0.989, 1.827) 0.059	1.393(1.020, 1.903) 0.367	2.318 (1.317, 4.082) 0.004
Males			
Non-obesity	Reference	Reference	Reference
obesity	1.669(1.123, 2.482) 0.011	1.627(1.090, -2.429)0.017	2.713 (1.306, 5.638) 0.007
Females			
Non-obesity	Reference	Reference	Reference
obesity	0.987(0.602,1.621)0.960	1.136(0.685, -1.883)0.621	1.716 (0.647, 4.556) 0.278

**Notes:** Model 1:No covariates were adjusted. Model 2: Age, gender was adjusted. Model 3: Age, gender, educational level, ratio of family income to poverty, physical activity, Serum uric acid, Serum sodium, glycohemoglobin, fasting glucose, triglyceride, Total Cholesterol, HDL-Cholesterol, LDL-Cholesterol were adjusted.

### Associations of obesity status with 25OHD

As shown in Table 3, obesity status showed a negative association with 25-hydroxyvitamin D in all three models (model 1: β = -4.535, 95% CI: -6.987, -2.083; model 2 β = -4.249, 95% CI: -6.549, -2.039; model 3 β = -1.734, 95% CI: -7.285, 3.816). Further analysis by gender and adjustment for all confounders revealed that this negative association persisted among females (females: β = -4.091, 95% CI: -13.398, 5.217), but became positive among males (males: β = 1.833, 95% CI: -4.607, 8.273).

**Table 3:** Association between obesity status and 25-hydroxyvitamin D (nmol/L).

	Model 1 β (95%CI, P)	Model 2 β (95%CI, P)	Model 3 β (95%CI, P)
Non-obesity	Reference	Reference	Reference
obesity	-4.535(-6.987, -2.083) <0.001	-4.249(-6.549, -2.039) <0.001	-1.734 (-7.285, 3.816) 0.540
Males			
Non-obesity	Reference	Reference	Reference
obesity	-5.545(-8.483, -2.606) <0.001	-4.100(-6.826, -1.374)0.003	1.833 (-4.607, 8.273) 0.577
Females			
Non-obesity	Reference	Reference	Reference
obesity	-2.829(-6.679,1.021)0.150	-5.157(-8.613, -1.701)0.004	-4.091 (-13.398, 5.217) 0.389

**Notes:** Model 1:No covariates were adjusted. Model 2: Age, gender was adjusted. Model 3: Age, gender, educational level, ratio of family income to poverty, physical activity, Serum uric acid, Serum sodium, glycohemoglobin, fasting glucose, triglyceride, Total Cholesterol, HDL-Cholesterol, LDL-Cholesterol were adjusted.

### Associations of BMI with 25OHD

As shown in Table 4, there was no significant association between BMI and 25OHD in either gender, with or without obesity. This result remains consistent even after adjusting for potential confounders (males with obesity: β = -0.271, 95% CI: -2.449,1.907; females with obesity: β = 1.477, 95% CI: -1.486,4.440; males without obesity: β = 0.843, 95% CI: -1.010,2.696; females without obesity: β = -0.193, 95% CI: -2.388,2.001).

**Table 4:** Association between body mass index(kg/m^2^) and 25-hydroxyvitamin D (nmol/L).

	Model 1 β (95%CI, P)	Model 2 β (95%CI, P)	Model 3 β (95%CI, P)
Total	-0.414(-0.647, -0.181) <0.001	-0.441(-0.652, -0.230) <0.001	0.043 (-0.939, 1.026) 0.931
Males with obesity	-0.530(-1.178,0.117)0.109	-0.130(-0.744,0.484)0.679	-0.271 (-2.449, 1.907) 0.808
Females with obesity	-0.411(-1.320,0.499)0.377	-0.104(-0.921,0.714)0.804	1.477 (-1.486, 4.440) 0.330
Males without obesity	0.360(-0.288,1.007)0.276	-0.077(-0.679,0.525)0.802	0.843 (-1.010, 2.696) 0.373
Females without obesity	0.236 (-0.499, 0.971) 0.529	-0.887 (-1.558, -0.217)0.010	-0.193 (-2.388, 2.001) 0.863

**Notes:** Model 1:No covariates were adjusted. Model 2: Age, gender was adjusted. Model 3: Age, gender (not adjusted for in the subgroup analyses), educational level, ratio of family income to poverty, physical activity, Serum uric acid, Serum sodium, glycohemoglobin, fasting glucose, triglyceride, Total Cholesterol, HDL-Cholesterol and LDL-Cholesterol were adjusted.

### Association of WC with 25OHD

As shown in Table 5, when all confounding variables were taken into account, there was a negative correlation between WC and 25OHD (β = -0.587, 95% CI: -0.974, -0.201). After stratifying by gender and obesity status, we found that the relationship between WC and 25OHD remained significantly negatively correlated for male participants regardless of their obesity status (males with obesity: β = -1.461, 95% CI: -2.485, -0.436; males without obesity: β = -0.855, 95% CI: -1.499, -0.210), while for female participants with or without obesity, there was no significant correlation between WC and 25OHD (females with obesity: β = 0.086, 95% CI: -0.798, 0.970; females without obesity: β = -0.365, 95% CI: -1.098, 0.369).

**Table 5:** Association between waist circumference (cm) and 25-hydroxyvitamin D (nmol/L).

	Model 1 β (95%CI, P)	Model 2 β (95%CI, P)	Model 3 β (95%CI, P)
Total	-0.093 (-0.185, 0.000) 0.051	-0.236 (-0.324, -0.147) <0.001	-0.587 (-0.974, -0.201) 0.003
Males with obesity	-0.201 (-0.471, 0.068) 0.144	-0.235 (-0.486, 0.015) 0.067	-1.461 (-2.485, -0.436) 0.006
Females with obesity	-0.073 (-0.442, 0.295) 0.696	-0.235 (-0.567, 0.097) 0.167	0.086 (-0.798, 0.970) 0.850
Males without obesity	0.274 (0.080, 0.469) 0.006	-0.195 (-0.391, -0.000) 0.050	-0.855 (-1.499, -0.210) 0.010
Females without obesity	0.475 (0.225, 0.725) <0.001	-0.278 (-0.524, -0.032) 0.027	-0.365 (-1.098, 0.369) 0.331

**Notes:** Model 1:No covariates were adjusted. Model 2: Age, gender was adjusted. Model 3: Age, gender (not adjusted for in the subgroup analyses), educational level, ratio of family income to poverty, physical activity, Serum uric acid, Serum sodium, glycohemoglobin, fasting glucose, triglyceride, Total Cholesterol, HDL-Cholesterol, LDL-Cholesterol were adjusted.

### Association of Weight with 25OHD

As shown in Table 6, after adjusting for all confounding factors, in obese males, there was a very strong positive connection between weight and 25OHD (β = 0.912, 95% CI: 0.227, 1.597). However, no significant correlations were found in other groups stratified by sex and obesity status (females with obesity: β = -0.412, 95% CI: -1.401, 0.577; males without obesity: β = 0.479, 95% CI: -0.029, 0.988; female without obesity: β = 0.031, 95% CI: -0.718, 0.781).

**Table 6:** Association between weight (kg) and 25-hydroxyvitamin D (nmol/L).

	Model 1β (95%CI, P)	Model 2β (95%CI, P)	Model 3β (95%CI, P)
Total	-0.288 (-0.358, -0.218) <0.001	-0.122 (-0.194, -0.049) 0.001	0.289 (-0.040, 0.619) 0.086
Males with obesity	-0.141 (-0.311, 0.029) 0.104	0.035 (-0.130, 0.201) 0.676	0.912 (0.227, 1.597) 0.010
Females with obesity	-0.379 (-0.679, -0.080) 0.014	-0.039 (-0.321, 0.242) 0.784	-0.412 (-1.401, 0.577) 0.416
Males without obesity	-0.129 (-0.298, 0.040) 0.135	0.022 (-0.136, 0.180) 0.786	0.479 (-0.029, 0.988) 0.066
Females without obesity	-0.330 (-0.598, -0.063) 0.016	-0.323 (-0.563, -0.084) 0.008	0.031 (-0.718, 0.781) 0.935

**Notes:** Model 1:No covariates were adjusted. Model 2: Age, gender was adjusted. Model 3: Age, gender (not adjusted for in the subgroup analyses), educational level, ratio of family income to poverty, physical activity, Serum uric acid, Serum sodium, glycohemoglobin, fasting glucose, triglyceride, Total Cholesterol, HDL-Cholesterol, LDL-Cholesterol were adjusted.

## Discussion

This study examined the relationship between obesity, BMI, WC, and body weight in Asian people and 25OHD. Our findings suggest that there are gender differences in the correlation between obesity and 25OHD. Additionally, we observed a significant negative association between WC and 25OHD in among both obese and non-obese men. Furthermore, weight was significantly more positively correlated with 25OHD in obese men compared to non-obese patients.

Obesity and vitamin D deficiency have evolved into global pandemics. Our findings suggested a negative association between obesity and vitamin D, which was consistent with previous studies reporting lower serum 25-hydroxyvitamin D in obese subjects than in non-obese subjects [22,23]. Most of the vitamin D is primarily formed through UVB (wavelength 290-315mm) exposure to the skin, with a smaller portion coming from dietary sources such as liver, milk, and dietary supplements [24,25]. Several studies have suggested that low concentrations of 25OHD in obese individuals may be associated with less outdoor exercise, lower sun exposure and dietary preferences [13,26,27]. Another study suggests that vitamin D is mainly stored in adipose tissue in obese individuals, leading to low concentrations of circulating 25OHD [28]. Notably, we discovered that the link between obesity and 25OHD varied across men and women after controlling for confounders, which leads us to believe that sex hormones may have played a role. In studies in Korea and Serbia, obese women were found to be more likely to suffer from vitamin D deficiency [29,30]. However, two studies from India and Norway showed that obese men had a considerably higher frequency of vitamin D deficiency than thin men [31,32]. Besides, another study on Bahraini adults showed that 25OHD levels were significantly higher in obese men than in women [33]. The findings of these studies are contradictory. One possible reason for this is the differences in race, ethnicity, and sample size of the included populations. To determine if there is a specific gender linked to obesity and vitamin D, we need an RCT research.

WC is a simple and clinically applicable indicator for determining abdominal obesity. It has been significantly linked to both cardiovascular and all-cause mortality [34–37]. In clinical practice, waist circumference can provide additional evidence to predict higher risk phenotypes for obesity [38]. A previous meta-analysis study found a direct correlation between waist circumference and risk of vitamin D deficiency, and this correlation persisted in a subgroup analysis by sex [9]. However, our research revealed a significant negative association between WC and 25OHD only in Asian adult males. This phenomenon was even more pronounced in men with obesity. One possible reason is that Asians have a higher tendency to deposit fat in their internal organs than Europeans, making them more prone to abdominal obesity [39,40], Men are more likely than women to accumulate fat in the abdomen [41]. According to a 2011–2018 NHANES research on obesity trends by race, there has been an alarming rise in body fat percentage among non-Hispanic Asians, particularly men. The incidence of abdominal obesity among this group increased by 29.1% during this period [2]. Another possible reason for the negative association could be low total testosterone levels obeserved in obese men [42–44]. Growing evidence suggested that testosterone can affect vitamin D metabolism, and low levels of testosterone can lead to decreased concentrations of 25OHD [45,46]. This is another study that seems to add to the growing literature that shows WC to be more sensitive at assessing co-morbidities of obesity than BMI.

Obesity can be visually observed through a patient’s weight. High body weight may cause a decrease in vitamin D levels, but studies have shown conflicting results regarding the relationship between vitamin D status and weight gain. A previous meta-analysis study found that weight and fat loss increased circulating 25OHD concentrations [10], while a prospective cohort study in Switzerland showed no correlation between vitamin D status and weight gain [12]. After stratifying by obesity status and gender and controlling for confounding factors, it was found that weight was significantly and positively associated with 25OHD in obese men. The contradictory results among studies may be due to the heterogeneity between research, including changes in population selection, the method used to quantify 25OHD, sample size, and controlled confounders.

NHANES is a nationally representative dataset that we used in our study. We primarily derived most of the variables from objective data on anthropometric and laboratory outcomes to ensure a higher level of confidence in our results. Additionally, we used a sufficiently large sample size to identify non-Hispanic Asians, which allowed us to better understand the relationship between obesity and vitamin D—this is the main advantage of our study. Nevertheless, our research has certain limitations as well. First, since this study was cross-sectional, we were unable to draw any conclusions about the cause of the link between obesity and vitamin D. Second, due to the limitations of NHANES data collection, we had no access to important variables affecting vitamin D, such as regional latitude, season and outdoor activity. Third, using BMI alone may lead to bias when determining obesity for muscular athletes or elderly people. Fourth, physical activity status and some of the covariates were generated through self-reporting, which may have resulted in reporting bias.

## Conclusion

In conclusion, this research discovered a link between vitamin D and obesity in non-Hispanic Asian populations. The findings highlight a significant negative association between waist circumference and vitamin D, particularly among obese males. These findings suggest that reducing waist circumference may help alleviate vitamin D deficiency in obese men. However, further prospective cohort studies are necessary to validate these benefits.

## Supporting information

S1 Data (XLS)
